# On the application of the tolerance factor to inorganic and hybrid halide perovskites: a revised system†

**DOI:** 10.1039/c5sc04845a

**Published:** 2016-04-01

**Authors:** W. Travis, E. N. K. Glover, H. Bronstein, D. O. Scanlon, R. G. Palgrave

**Affiliations:** a Department of Chemistry, University College London 20 Gordon Street London WC1H 0AJ UK r.palgrave@ucl.ac.uk; b University College London, Kathleen Lonsdale Materials Chemistry, Department of Chemistry 20 Gordon Street London WC1H 0AJ UK; c Diamond Light Source Ltd., Diamond House Harwell Science and Innovation Campus Didcot Oxfordshire OX11 0DE UK

## Abstract

The tolerance factor is a widely used predictor of perovskite stability. The recent interest in hybrid perovskites for use as solar cell absorbers has lead to application of the tolerance factor to these materials as a way to explain and predict structure. Here we critically assess the suitability of the tolerance factor for halide perovskites. We show that the tolerance factor fails to accurately predict the stability of the 32 known inorganic iodide perovskites, and propose an alternative method. We introduce a revised set of ionic radii for cations that is anion dependent, this revision is necessary due to increased covalency in metal–halide bonds for heavier halides compared with the metal-oxide and fluoride bonds used to calculate Shannon radii. We also employ a 2D structural map to account for the size requirements of the halide anions. Together these measures yield a simple system which may assist in the search for new hybrid and inorganic perovskites.

## Abstract 2

Can new hybrid perovskites be predicted using the tolerance factor?

## Graphical abstract

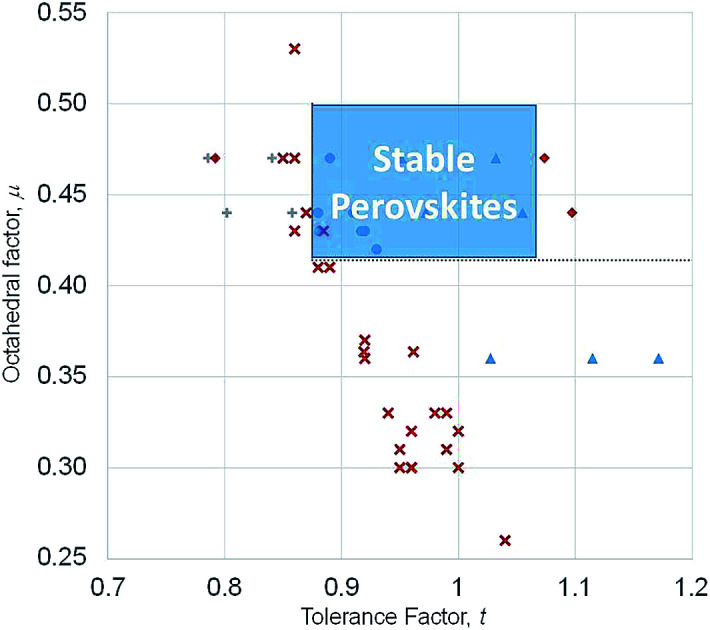

## Introduction

Predicting the most stable structure for a given chemical composition is an ongoing challenge in chemistry, particularly for solid state non-molecular inorganic compounds. The advent of modern computational methods has significantly advanced our ability to successfully predict the structure of a previously unknown composition.^1,2^ These computational approaches, however, remain time intensive, and are not suitable for all compounds. In contrast, simple geometric approaches to the understanding and prediction of stability in ionic solid state structures have been used for around a century. In such approaches, the constituent ions are assumed to be hard spheres, and, following the methodology of Shannon,^3,4^ their radii can be assumed, with remarkable success, to be constant for a given charge state and coordination number. A simple calculation of ratios of ionic radii can assess whether spheres of a particular size can pack together in a particular structure. The perovskite structure is one of the most widely studied solid state structures, and its understanding has been greatly aided by the use of geometric approaches.

The perovskite structure can be adopted by compounds of formula ABX_3_, where A and B are cations and X is an anion. It is based on a cubic array of corner sharing BX_6_ octahedra, with the A site cation located within the cuboctahedral cavities. An alternative way to view the structure is of a cubic close packed AX_3_ array with the B site cations within the octahedral holes. For the perovskite structure, the most commonly used and most successful geometric ratio is the Goldschmidt tolerance factor, *t*, defined as follows:^5^1
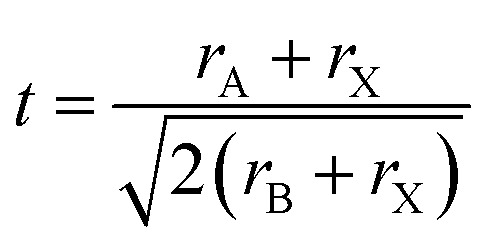
where *r*_A_ and *r*_B_ are the ionic radius of the A and B site cations respectively, and *r*_X_ is the ionic radius of the anion. The tolerance factor assesses whether the A site cation can fit within the cavities in the BX_3_ framework. A tolerance factor of 1 indicates a perfect fit; in the range 0.8 ≤ *t* ≤ 1 perovskites generally do form, although in the lower part of this range they may be distorted due to tilting of the BX_6_ octahedra and lowering of the symmetry. If *t* > 1, this indicates the A site cation is too large and generally precludes formation of a perovskite, and if *t* < 0.8, the A cation is too small, again often leading to alternative structures. The tolerance factor has been very successful in describing and predicting oxide and fluoride perovskite stability, *i.e.* ABX_3_ compounds where X = O^2−^ or F^−^.^6^ In these compounds, the high electronegativity of the anions leads to a large degree of ionicity in the bonding, and makes the assumptions of the hard sphere model more valid. In their 2004 review of ABO_3_ compounds, Li *et al.* identified 192 ABO_3_ compounds, of which 121 formed perovskites at room temperature and pressure.^7^ Out of 192 compounds, 163 (85%) were categorised correctly as perovskites or non-perovskites using the tolerance factor criterion for perovskite stability 0.8 ≤ *t* ≤ 1. The same authors also found that for 65 ABF_3_ compounds, 62 (95%) were correctly classified using a criterion for perovskite stability of *t* > 0.85.^8^

Recently, halide perovskites have attracted very significant attention due to the emergence of hybrid perovskite solar absorbers after an initial report in 2009 by Kojima *et al.*^9^ Hybrid halide perovskites are compounds of formula ABX_3_ where the A site is occupied by a small organic cation, such as methylammonium, CH_3_NH_3_^+^ and X is a halide anion. These materials are able to absorb light and separate the resulting charge carriers with remarkable efficiency, yet can be produced through simple, bench top chemistry, and are presently the most exciting of the emerging solar cell technologies.^10–14^ The most efficient hybrid solar cell materials use iodide on the X site of a perovskite structure, as this leads to a band gap close to the optimum for single junction PV cells. CH_3_NH_3_PbI_3_ has a band gap of around 1.5 eV and is the most promising of the hybrid PV solar absorber materials.^15^ Substituting iodide with lighter halides, or adoption of alternative ABX_3_ structures such as hexagonal perovskite, both lead to a widening of the bandgap and concurrent decrease of the PV efficiency.^16^ Prior to the discovery of hybrid solar cells, hybrid iodide perovskites and indeed purely inorganic iodide perovskites had been studied for many years in relation to several diverse applications.^17–24^

Due to the very rapid and dramatic advance of the hybrid perovskite solar cell field, there is a great motivation to find new hybrid halide perovskite compounds,^12,25^ and in order to predict compositions that will form stable perovskite structures, the tolerance factor has been widely employed.^26–28^ However, caution is necessary, as a number of the assumptions underlying any geometric approach to predicting solid state structures must be questioned for the case of the hybrid halide perovskites:

(1) The organic cations are non-spherical, and so an obvious difficulty is encountered in defining the A site ionic radius for use in eqn (1).

(2) Due to the low decomposition temperatures of the organic molecular ions that occupy the A site, hybrid perovskites tend to be produced using low temperature syntheses, meaning that kinetic trapping of less thermodynamically stable structures is possible.

(3) The lower electronegativity of the heavier halides and greater chemical softness, especially of the iodide anion, compared with oxides and fluorides means that the assumption that the ions are unpolarisable hard spheres is less valid.

(4) The tables of cation ionic radii composed by Shannon *et al.* are taken from oxide and fluoride compounds only, therefore it necessary to question how well they apply to the heavier halides.

The severity of points 3 and 4 is expected to increase moving from chloride to iodide anions. Yet it is the iodides (and to some extent bromides) which are of current technological interest as PV absorber materials, because, as already mentioned, the heavier halides result in compounds with optimal band gaps for solar absorption. Predicting the stability of the heavier hybrid halide perovskites is of most pressing need, and is also expected to be most challenging given the chemical differences between the heavier and lighter halides mentioned in points 3 and 4 above.

For this reason we will first critically examine the applicability of tolerance factor and other geometric criteria to the prediction of hybrid iodide perovskite stability. Cheetham and co-workers have suggested that the range of stability for hybrid iodide perovskites is roughly 0.8 ≤ *t* ≤ 1, *i.e.* very similar to that found for the oxides and fluorides.^26,27^ This makes intuitive sense as the stability limits are based on geometry rather than any chemical properties, so in principle might be assumed to be universal. We note that whilst points 1 and to an extent 2 (above) apply specifically to hybrid perovskites, points 3 and 4 apply equally to inorganic iodide perovskites too, *i.e.* compounds in which A is a simple inorganic cation. As a starting point, therefore, we have tested the tolerance factor criterion 0.8 ≤ *t* ≤ 1 against the known inorganic ABI_3_ compounds. A search of the Inorganic Crystal Structure Database (ICSD), supplemented by a general literature search, revealed 32 crystallographically characterised inorganic ABI_3_ compounds. Of these, eight formed perovskite structures at room temperature and pressure (RbDyI_3_, RbTmI_3_, CsCaI_3_, RbSnI_3_, CsSnI_3_, CsDyI_3_, CsYbI_3_, CsPbI_3_),^21–23,29–36^ whilst 24 did not (see ESI Table S3† for a lookup table of ABI_3_ references sorted by A and B cation). Compounds are categorised as a perovskites if the structure is based on a cubic close packed AX_3_ sublattice, *i.e.* their BX_3_ sublattice consists exclusively of corner sharing octahedra connected in three dimensions. The prototypical perovskite structure in the *Pm*3̄*m* space group, and structures related to this through tilting of the BX_6_ octahedra or off centring of the A site cation are therefore included as perovskites. Compounds commonly referred to as hexagonal perovskites, where some proportion of the AX_3_ sublattice adopts hexagonal close packing, and where some degree of BX_6_ octahedra edge sharing is present, are listed as non-perovskites for the purposes of this discussion, since these compounds exhibit much larger band gaps than their perovskite counterparts, making them generally unsuitable for PV applications.

**Fig. 1:**
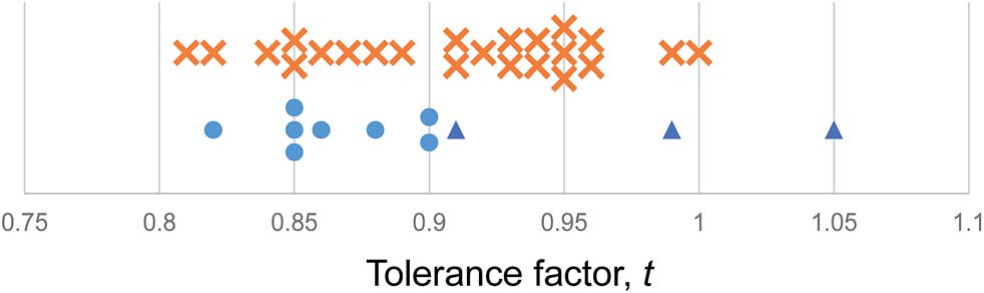
The tolerance factors of ABI_3_ compounds. Blue dots represent inorganic ABI_3_ compounds that form perovskites at room temperature and pressure. Red crosses represent inorganic ABI_3_ compounds that do not form perovskites. Blue triangles show hybrid APbI_3_ and ASnI_3_ compounds that form in the perovskite structure, using the methodology of Cheetham *et al.* to estimate molecular ion radii.^26,27^ There is no boundary on the tolerance factor scale that separates perovskites from non-perovskites.

We use the Shannon radii as employed by Cheetham *et al.* and others^8,26,27^ to calculate *t* for each of the known purely inorganic ABI_3_ compounds. Fig. 1 shows the distribution of tolerance factors, *t*, calculated for these compounds and expressed to two decimal places. Blue dots represent perovskites and red crosses represent non perovskites. As can be seen, all 32 compounds fall within the range 0.8 ≤ *t* ≤ 1, so we conclude that this criterion is not useful for predicting or explaining structure stability.

Using the tolerance factor alone, the best empirical criterion that can be applied to this set of known inorganic ABI_3_ compounds is perovskite stability in the range 0.8 ≤ *t* ≤ 0.9. Within this range, all eight of the known perovskite compounds are found. However, nine out of 24 non perovskites are also in this region. Thus using a perovskite stability criterion of 0.8 ≤ *t* ≤ 0.9, 72% of inorganic ABI_3_ compounds are categorised correctly. This figure is lower than can be achieved for the oxides and fluorides, but furthermore this second criterion is less satisfying, as when hybrid compounds are considered, which have large A site cations and therefore large tolerance factors, such a criterion would predict that no hybrid perovskites are stable, which is not the case.

We conclude from the above analysis that having a tolerance factor within a specific range (calculated from traditional Shannon radii) may be a necessary condition for perovskite formation but it is not a sufficient condition, and the traditional approach that works reasonably effectively for fluoride and oxide compounds cannot be used to explain the known structures of the inorganic ABI_3_ compounds. Given the additional difficulties pertinent to prediction of hybrid structures, over and above those encountered for the purely inorganic iodides, we suggest that there is no reason to expect this approach as it stands would be successful in predicting the stability or otherwise new hybrid perovskite structures.

A question then presents itself: is it possible to use geometric methods to understand and predict halide perovskite stability in general and hybrid iodide perovskite stability in particular? Here we cautiously answer in the affirmative by introducing an adapted approach that takes into account the chemical and physical differences between the heavier halides and the fluoride and oxide anions for which the tolerance factor approach is successful. Using this approach we are able to draw a structure map with simple criteria for halide perovskite stability that gives a success rate over 92% for the library of inorganic halide perovskites complied by Li *et al.*^8^ The hybrid perovskites present further challenges but broadly fit within the stability framework of their inorganic counterparts. We believe the concepts we set out below could be extended to other halide structures as well.

## Approach

Structures containing chloride, bromide or iodide anions have several important chemical differences compared with fluorides or oxides for which the concept of Shannon radii and tolerance factors were originally developed. Firstly, the anion is now larger. The iodide anion has a Shannon radius of 2.20 Å, compared to 1.28 Å for fluoride and 1.35 Å for oxide. Secondly, the heavier halides are less electronegative: I is 2.66 on the Pauling electronegativity scale compared with O at 3.16 and F at 3.98. This means bonds between heavier halides and metals will tend to have greater covalency, which should increase down the halogen group, and the model of hard spheres will be less applicable. We propose two main adaptations that are necessary from the procedure used with oxides and fluorides.

Each of these points will be elaborated on below:

(1) Revised cation radii: for heavier halides, a different set of cation ionic radii must be used for the p, d and f block metals to account for the deviations from Shannon radii.

(2) Additional geometric considerations: a suitable tolerance factor is a necessary but not a sufficient condition for formation of the perovskite structure by ABX_3_ compounds, where X = Cl, Br, I. This is due to the larger size of the anion which makes other geometric considerations, especially the ability to octahedrally coordinate a given metal cation, of equal importance to the tolerance factor in determining perovskite stability.

### Revised cation radii

A point not always noted is that the widely used Shannon cation radii are calculated from oxide and fluoride compounds only.^3,4^ Less electronegative anions will result in a greater degree of covalency in the metal–anion bonds; this phenomenon was recognised and quantified by Shannon and co-authors in a series of papers by using a *covalency parameter* to indicate deviation from ‘pure’ ionic bonding.^3,37,38^ The influence of increasing covalency is that observed bond lengths are expected to be shorter than the sum of the two Shannon radii. The sum of the appropriate Shannon radii for a given bond shall henceforth be referred to here as the Shannon bond length, *D*_Shannon_. For example, the Pb–F Shannon bond length for octahedrally coordinated Pb is *D*_Shannon_(Pb–F) = *r*_Pb(II)_ + *r*_F^−^_ = 1.19 + 1.285 = 2.475 Å. If the variation in experimental interatomic distances compared to the Shannon bond length is due in whole or in part to increased covalency, then it is expected to be seen most prominently for the less electropositive metals of the p and d block, as for these compounds the difference in electronegativities between metal and anion, Δ*χ*, is small, so the degree of covalency is greater. The effect would be smaller for the s and f block metals.

To assess the applicability of Shannon radii to non-oxide/fluoride compounds, and to quantify any deviation from *D*_Shannon_ upon moving to heavier anions, a general survey was undertaken of experimental bond lengths in metal halide compounds, not limited to perovskite compounds. Given that the motivation for this work is predicting stable halide perovskite structures, we consider M–X bond lengths where the metal M is a candidate for the B site of the ABX_3_ halide perovskite structure. The perovskite B site is octahedrally coordinated by halide anions, so we limit our search to compounds containing MX_6_ octahedra, where M is a metal from a selection to be defined shortly, and X is a halide. The list of metals M to be considered was limited to those that could feasibly occupy the B site of such a perovskite, *i.e.* divalent metals for which AMX_3_ compounds are known and crystallographically characterised. This list of metals, M, used in this work is: Mg, Ca, Ti, V, Cr, Mn, Fe, Ni, Cd, Hg, Ge, Sn, Pb, Sm, Yb, Dy, Tm. Experimental room temperature and pressure structures were obtained from the ICSD using the CrystalWorks software. All compounds that include divalent metals from the list above, coordinated with exactly six of the same halide anions (and no other anions) were included. A total of 579 compounds were found matching the criteria set out above. Metal–halide distances for the first coordination sphere of the metal were calculated from the crystallographic information files. The experimental bond length, *D*_obs_(M–X), was then taken as the mean of these metal–anion distances. Table S1 in the ESI† shows the number of compounds used for calculation of the bond length for each metal–halide pair, and the standard deviation of bond lengths for each bond.

**Fig. 2:**
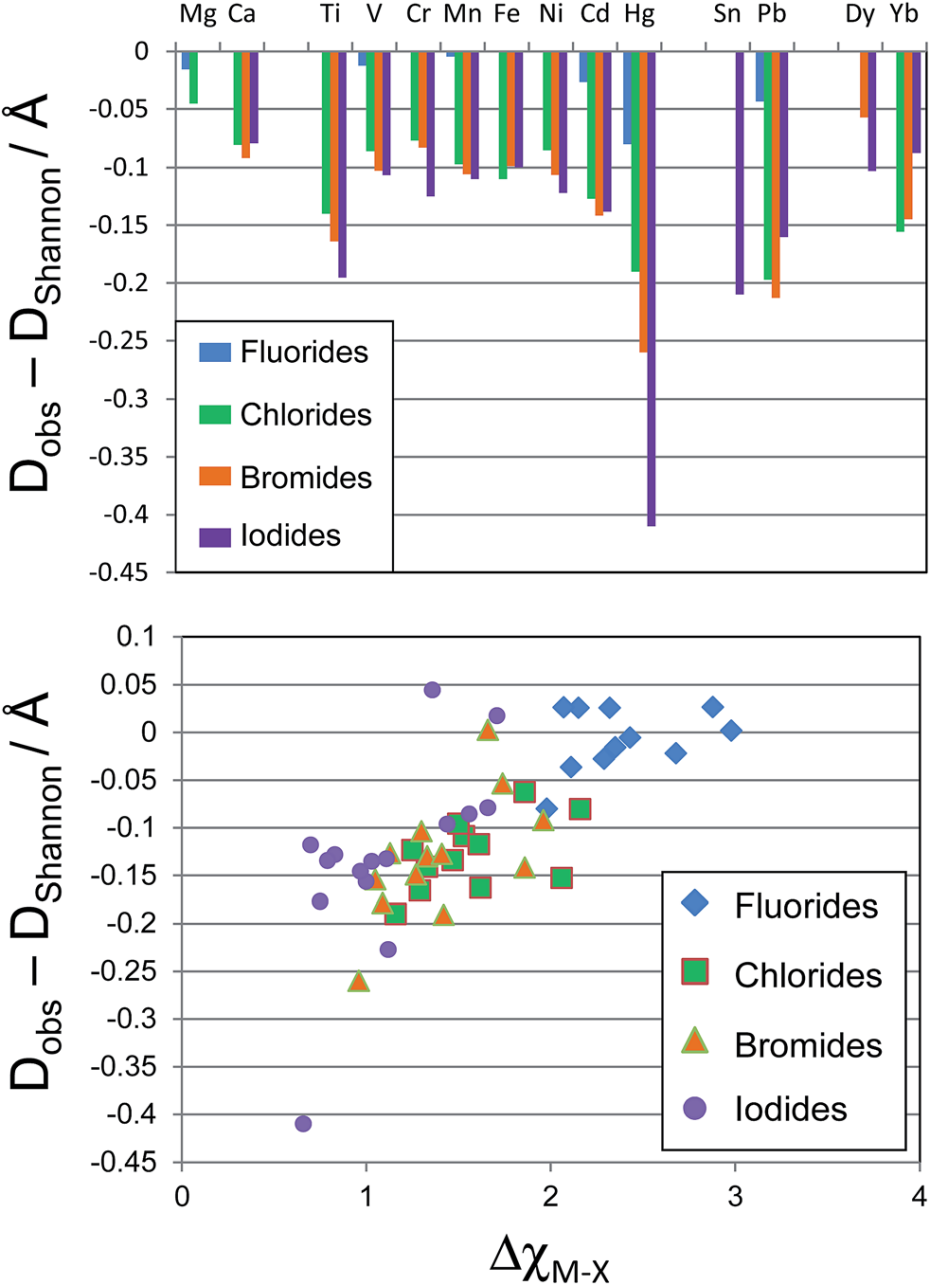
Top, chart of deviation in experimental bond lengths from Shannon bond lengths in octahedral M–X bonds (X = halide) by element. Bottom, plot of deviation in experimental bond lengths from Shannon bond lengths against difference in electronegativity.

The deviation of *D*_obs_(M–X) from *D*_Shannon_ was calculated and is plotted in Fig. 2. The Shannon bond length very closely approximates the measured bond lengths for all of the metal fluoride compounds considered. The Hg–F bond showed the largest deviation of any metal considered here, the average Hg–F bond distance was measured as 0.08 Å shorter than the Hg–F Shannon bond length, while all of the first row transition metal fluoride bonds were within 0.02 Å of the Shannon bond length.

However, for X = Cl, Br and I the M–X bonds for all metals considered (except Mg) show considerable variation from the Shannon bond lengths. The d block metals typically show a shortening of their observed M–X bond lengths compared with the Shannon bond length by 0.075–0.1 Å, with Ti, Cd and Hg showing a considerably greater variation. For the d block metals the deviation tends to increase moving down the halide group from Cl to I. The p block post transition metals also show significant deviation from the Shannon bond lengths. Average Pb–Br bonds are observed to be 0.21 Å shorter than the Pb–Br Shannon bond length. Sn–I bonds show a similar deviation from expected values.

If the deviation from Shannon bond length is due to increased covalency as has been suggested, then the effects should scale with the difference in electronegativity between the metal and halogen, Δ*χ*_M–X_, as a smaller electronegativity difference tends to greater covalency. Fig. 2 shows a plot of Δ*χ*_M–X_ against deviation from Shannon bond length for all M–X bonds considered above. As can be seen, there is a correlation between the two variables, with smaller Δ*χ*_M–X_ values tending to result in larger deviation from Shannon bond lengths. This suggests that the deviations are at least partially due to increased covalency in the M–X bonds for the heavier halides. Some M–X bonds do not seem to follow the general trend. For example the Mg–I and Mg–Br experimental bond length shows almost no variation from the Shannon bond length. This may be due to the chemical hardness of Mg^2+^, which is considerably greater than for other elements considered here: the Pearson hardness for Mg^2+^, *η* = 32.55 eV, while all of the d-block metals considered here have *η* < 10.3 eV.^39^ The Hg–X bonds, however, show exceptionally large deviations: the Hg–I bond length is over 0.4 Å shorter than the Shannon bond length. This is despite the Pearson hardness of Hg^2+^ being close to those of the other d block metals considered here.

These deviations from the expected interatomic distances, howsoever caused, will influence the calculation of geometric ratios used to assess structure stability. Since the effect of contraction of bond lengths is both anion and cation dependent, it is not sufficient to apply an overall corrective factor to the existing Shannon radii, or to adjust the tolerance factor stability limits to account for the contraction. It is proposed here that for geometric calculations on heavier halide (Cl, Br, I) structures, a modified set of ionic radii be used. For our purposes of understanding the structural stability of halide perovskites, it is convenient to maintain the ionic radius of the halide anions at its standard Shannon value and introduce a new set of cation radii for metals in halide compounds, *r*_M(X)_, which depends, like the Shannon radii, on the metal, the charge state, the coordination environment, but unlike the Shannon radii, also on the halide to which the metal is bonded. These revised ratios are shown in Table 1, together with the corresponding Shannon radii. To obtain *r*_M(X)_ values, the Shannon ionic radius of the appropriate halide anion (1.285 Å for fluoride, 1.85 Å for chloride, 1.96 Å for bromide, and 2.20 Å for iodide) was subtracted from the average experimental bond length to yield the cation radius for each metal.

**Table 1:** Revised ionic radii used for halide compounds, compared with the corresponding Shannon radii.^3^ HS = high spin

Cation	Six coordinate Shannon ionic radius/Å	Experimental 6-coordinate cation radius, *r*_M(X)_			
		Fluoride compounds/Å	Chloride compounds/Å	Bromide compounds/Å	Iodide compounds/Å
Mg(ii)	0.72	0.70	0.67	0.72	0.75^a^
Ca(ii)	1.00	1.00	0.92^a^	0.91^a^	0.92
Sr(ii)	1.16	—	—	—	1.18
Ti(ii)	0.86	—	0.72	0.70	0.66
V(ii)	0.79	0.78	0.70	0.69	0.68
Cr(ii)	0.80(HS)	0.82	0.72^a^	0.72^a^	0.68
Mn(ii)	0.83(HS)	0.83	0.73	0.72	0.72
Fe(ii)	0.78(HS)	0.80	0.67^a^	0.68	0.68^a^
Ni(ii)	0.69	0.71	0.60	0.58	0.57^a^
Cd(ii)	0.95	0.92^a^	0.82	0.81	0.81
Hg(ii)	1.02	0.94	0.83^a^	0.76	0.61^a^
Ge(ii)	0.73	b	b	b	0.77
Sn(ii)	1.15^c^	—	—	—	0.97
Pb(ii)	1.19	1.15	0.99^a^	0.98	1.03
Tm(ii)	1.03	—	0.93^a^	—	0.95
Sm(ii)	1.22^d^	1.20^a^	1.02^a^	0.86	1.11^a^
Yb(ii)	1.02	1.05	0.86	0.88	0.93
Dy(ii)	1.07	—	—	1.01	0.97

a Less than three crystallographically characterised compounds found, or standard deviation of experimental bond lengths above 0.1 Å.

b Ge(ii) adopts highly distorted coordination environments so ionic radii not considered for F, Cl, Br.

c Shannon does not give a Sn(ii) radius, yet 1.15 Å has been used by others.^27^

d No 6-coordinate Sm(ii) radius is given by Shannon – the radius here is for 7 coordinate Sm(ii).^3,4^ For statistical analysis see the ESI.

### Additional geometric considerations

The tolerance factor alone, whether calculated using the revised radii in Table 1 or indeed the standard Shannon radii, is not sufficient for predicting the structures adopted by inorganic ABX_3_ compounds. In the following discussion, geometric ratios are calculated using the revised radii shown in Table 1 for the B site cations. The tolerance factor assesses whether the A cation can fit within the BX_3_ framework of corner sharing octahedra (referred to as the ReO_3_ structure) that is found in the cubic perovskite. However, another important consideration is whether the B site cation is of the correct size to be coordinated by six anions; *i.e.* whether the B site cation can fit in the octahedral hole in the anion sublattice. The radius of an octahedral hole, *r*_hole_ formed within six close packed spheres of radius *r* is:

2 *r*_hole_ = 0.41*r*

Therefore in the perovskite structure, assuming the hard sphere model for the ions, B site cations with radius smaller than 0.41*r*_X_ cannot be coordinated octahedrally without the anions overlapping. For the oxide and fluoride perovskites, the radius of the octahedral cavity (0.55 Å and 0.52 Å respectively) is such that only a few cations, for example P^5+^, As^5+^, and Si^4+^ are too small to fit within, and these cations are never found on the B site of oxide or fluoride perovskites. However, the octahedral cavity formed by six iodide anions is 0.90 Å in radius, and many potential B site cations are smaller than this, as can be seen from Table 1. To assess the fit of the B site cation into the X_6_ octahedron, several authors have utilised the octahedral factor *μ* defined as:^8^3
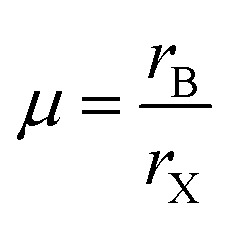


A plot of *t* against *μ* can then be constructed and used as a structure map. Such a map assesses the suitability of both the A site cation and the B site cation for the perovskite structure.

**Fig. 3:**
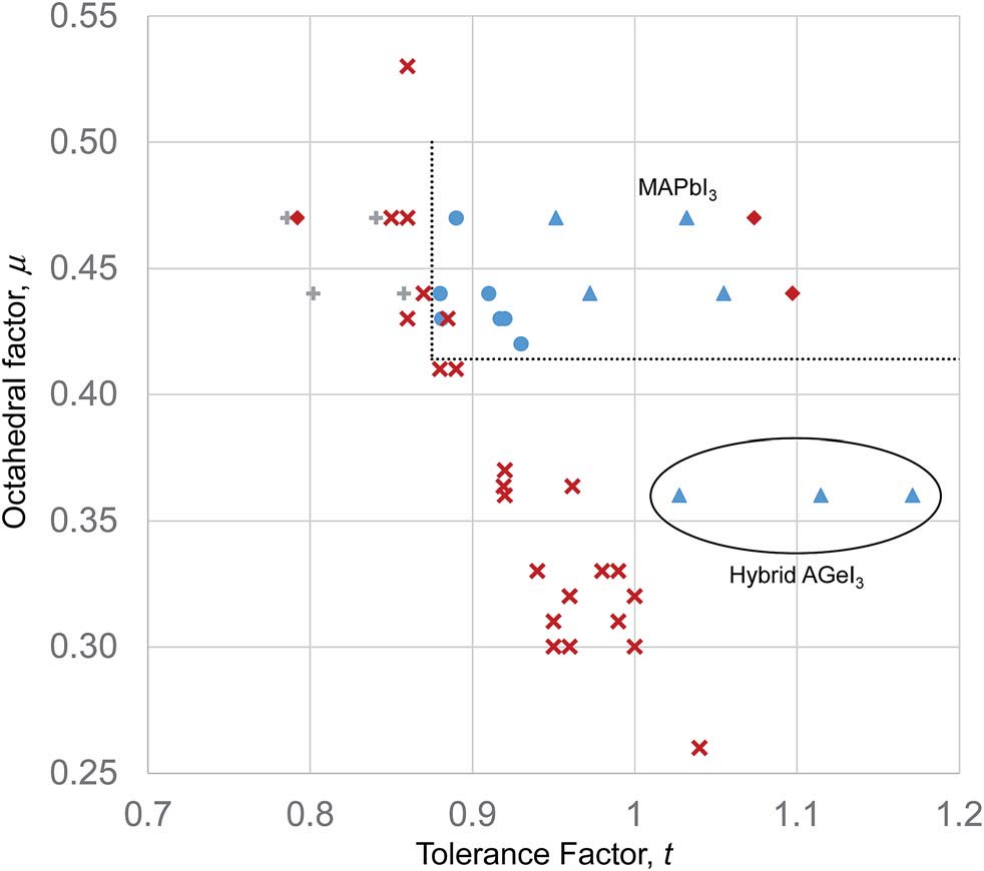
Structural map of ABI_3_ compounds. Blue dots represent stable inorganic perovskites. Red crosses represent inorganic compounds that do not form perovskites. Blue triangles represent stable hybrid perovskites, whilst red diamonds represent hybrid compositions that do not form perovskites. Dotted lines are the boundary lines mentioned in the text. The anomalous hybrid Ge(ii) compounds are highlighted, as is MAPbI_3_.

Such a *t*–*μ* plot for the iodide perovskites is shown in Fig. 3. This will be discussed in detail since the iodide perovskites are the focus of current attention as potential new PV absorber materials. It can be seen that inorganic iodide perovskites, represented by blue circles, form in a distinct region of the structure map. This region is bounded by two well defined lines. A lower horizontal boundary line is at constant octahedral factor, with perovskites forming when *μ* > 0.41. Below this line, all known inorganic ABI_3_ compositions adopt non-perovskite structures. This limit corresponds closely to that seen for ABO_3_ compounds (where the limit was found to be *μ* > 0.425).^7,40^ Furthermore, it is encouraging that the boundary line found for the iodides corresponds exactly to the geometric limit for octahedral coordination of the B site of *μ* = 0.41, as this suggests that the revised cation radii derived in Table 1 are appropriate for describing these structures. A vertical boundary line is *t* = 0.875. To the left (low tolerance factor) side of this line, six ABI_3_ compounds have been reported (RbPbI_3_, RbSnI_3_, KTmI_3_, TlPbI_3_, CsSrI_3_ and NH_4_PbI_3_), and none of these form perovskites. As well as these non perovskites, several points representing examples of unknown or uncharacterised compositions made up of common elements (NaPbI_3_, KSnI_3_, NaSnI_3_) are found to the left of this line, and are included on Fig. 3. The compound KPbI_3_, has been reported by several groups but no structure has been obtained, and there is uncertainty about the composition and even the colour.^41–43^ Therefore for the present this point is also marked as unknown.

The upper boundary in *μ*, should there be one, is not well defined. There is only one reported ABI_3_ compound (CsSrI_3_) which has *μ* > 0.47, and this is found at *μ* = 0.53, but in any case lies to the left of the vertical boundary line. Further synthetic efforts to produce compounds with higher octahedral factor are necessary to explore this region of the structure map and establish the true stability limits here.

The boundary of the stable perovskite region to the high tolerance factor side (right hand side as drawn in Fig. 1) is also not well defined, as there are no inorganic compounds with *t* > 0.92 and *μ* > 0.41. To achieve a high tolerance factor, a large A site or a small B site cation is needed. The largest elemental cation in the periodic table (excluding radioactive elements) is Cs^+^, with radius 1.88 Å in 12 coordination. Even if Cs^+^ were combined with a hypothetical B site cation with exactly the radius necessary to meet the octahedral factor stability limit (*r*_B_ = 0.41*r*_iodide_ = 0.41 × 2.20 Å = 0.90 Å), the resulting compound would have a tolerance factor of only 0.93. A smaller B site would lead to an octahedral factor too low for formation of perovskites. Thus for simple inorganic perovskites with *μ* > 0.41, the maximum *t* achievable is 0.93, and to achieve a higher *t* we must use complex cations as will be discussed in the following section.

For the inorganic iodide perovskites, the structural map and boundary lines set out above can separate the set of 32 known inorganic ABI_3_ structures successfully into perovskites and non-perovskites with only one compound miss-assigned. RbYbI_3_ sits inside the stable region of the structure map but is reported experimentally as a non-perovskite. RBYbI_3_ is close to the boundary of the stable region, and it may be that improved accuracy of the revised ionic radii will correct this.

**Fig. 4:**
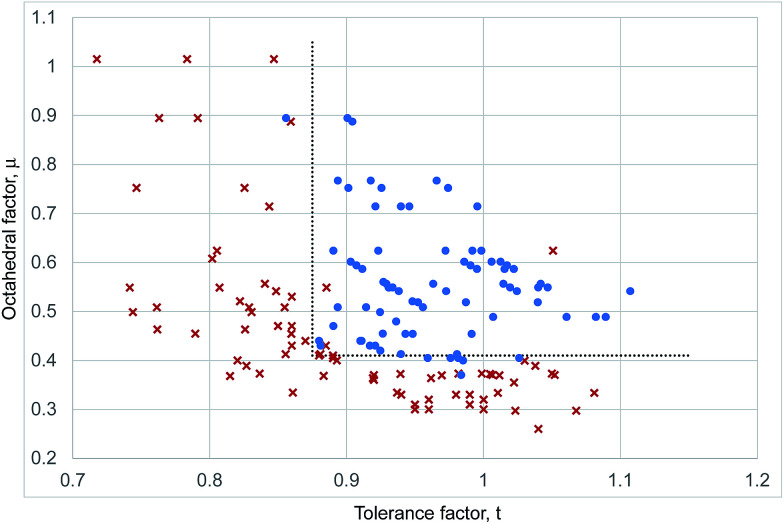
Structural map of inorganic ABX_3_ compounds. Blue dots represent inorganic compositions that adopt the perovskite structure at room temperature and pressure. Red crosses represent inorganic compounds that do not form perovskites. The dotted lines represent the boundaries of the stable perovskite region as described in the text.

Fig. 4 shows a combined structural map for all 159 halide perovskite considered here calculated with the revised cation radii from Table 1; the list of ABX_3_ compounds is that complied by Li *et al.*^8^ but treated with our revised ionic radii introduced above (Table S2, ESI†). The same stability criteria as used for the iodides, *μ* > 0.41 and *t* > 0.875, lead to 92% of the compositions considered being correctly determined as perovskites or non-perovskites. It can be seen that there is no defined upper boundary in octahedral factor, with examples of perovskite compounds with *μ* = 0.89, much higher than seen in the iodides.

Structural maps for the fluoride, chloride and bromide perovskites, constructed using the revised ionic radii from Table 1, are shown in the ESI (Fig. S1–S3†).

Based on octahedral factor criteria for perovskite stability, *μ* > 0.41, there are a limited number of cations that may occupy a B site for a particular halide perovskite. This is an essential consideration when determining whether a particular ABX_3_ compound will form a perovskite structure. Those cations with *r*_M(X)_ < 0.41*r*_X_ are too small to fit within the X_6_ octahedron. Table 2 shows a summary of the cations which give *μ* > 0.41 for each halide and therefore may be expected to be able to occupy the B site of fluoride, chloride, bromide and iodide perovskites. Since iodide has the largest *r*_X_ of the halides, only eight metals are sufficiently large to occupy the B site of an iodide perovskite: Pb, Sn, Yb, Dy, Tm, Sm, Ca, Sr. No inorganic A site cation is big enough to form ASrI_3_ or ASmI_3_ compounds with *t* > 0.875, so these are not predicted to form inorganic perovskites. There are three inorganic iodide compounds that have *t* and *μ* values within the stable perovskite region yet to our knowledge are so far unreported. These are TlDyI_3_, TlYbI_3_, and TlTmI_3_.

**Table 2:** List of metal cations that are predicted to be able to occupy the B site of halide perovskites, based on their revised anion dependent ionic radii listed in Table 1

Anion	Fluoride	Chloride	Bromide	Iodide
Size of octahedral hole = 0.41*r*_X_/Å	0.52	0.76	0.80	0.90
Divalent metal cations from Table 1 with *r*_M(X)_ > 0.41*r*_X_	All metals from Table 1	Ca, Cd, Hg, Pb, Sm, Tm, Yb	Ca, Cd, Pb, Yb, Dy, Sm	Ca, Sr, Pb, Sn, Yb, Dy, Sm, Tm

In summary, we demonstrate that an adapted geometric approach can categorise the room temperature structures of 31 out of 32 known inorganic iodide ABI_3_ compounds as perovskites or non-perovskites, and 147 correctly out of 159 ABX_3_ (X = F, Cl, Br, I) compositions. We introduce a revised set of cation radii for this task. These are anion specific and are calculated for divalent metals from the average six coordinate bond lengths of all compounds suitable compounds. The veracity of these cation radii is demonstrated in several ways. Firstly they deviate from Shannon radii in a manner consistent with the degree of covalency expected due to the electronegativity (Fig. 2). Secondly, they allow construction of a structural map for the halide perovskites with stability limits based on geometric principles, specifically the octahedral factor limit which coincides with the geometric size of a hole within six close packed halide ions, with one single set of stability criteria for all halide perovskites.

## Application to hybrid iodide perovskites

Hybrid perovskites as discussed here consist of an organic (usually a substituted ammonium) cation on the A site. In this discussion, we will include the ammonium ion (NH_4_^+^) itself along with organic A groups, although it is usually considered an inorganic ion. The hybrid iodide perovskites have recently become of great interest due to their exceptional photovoltaic properties. The following molecular cations have been successfully placed on the A site of an iodide perovskite: methylammonium (CH_3_NH_3_, abbreviated here as MA^+^),^11^ formamidinium (H_2_N–CH=NH_2_, FA^+^)^44^ and acetamidinium (CH_3_C(NH_2_)_2_^+^, AC^+^).^45^ From these, the following hybrid iodide perovskites with a single A site cation have been crystallographically characterised: MAPbI_3_, MASnI_3_, MAGeI_3_, FAPbI_3_, FASnI_3_, FAGeI_3_ and ACGeI_3_ (see Table S4 in ESI† for references for all iodide compounds). Mitzi has mentioned the successful synthesis of MAEuI_3_ (and CsEuI_3_), forming the perovskite structure^46^ but no crystallographic information could be found. Structures are known with mixed FA^+^ and MA^+^ cations on the A site.^44^ These would have *t* and *μ* values intermediate between the pure A site compounds and are not considered further here.

The use of geometric ratios and stability maps is more challenging for hybrid perovskites than pure inorganic perovskites for a number of reasons which have already been discussed. In addition to the difficulties in representing the chemistry of these materials using geometric models, a further issue is that confidence in any system of structure prediction depends on a large number of data points. Only a small number of hybrid materials in the perovskite structure have been characterised, so it is difficult to assess the validity of any proposed system; a very large number of stability criteria will give perfect categorisation of the few existing materials, but that does not necessarily imply any physical basis for the criteria or any predictive power for unknown structures.

We now turn to the issue of quantifying the size of the A site cation in hybrid materials. Several approaches have been taken to model the size of molecular cations. Cheetham *et al.* quantified ammonium molecular ion radii, *r*_Aeff_

4 *r*_Aeff_ = *r*_mass_ + *r*_ion_

where *r*_mass_ is the distance from the centre of mass of the molecule to the furthest non-hydrogen atom in the molecule, and *r*_ion_ is the Shannon ionic radius of the nitride (N^3−^) anion, which is 1.46 Å.^26^ The resulting ionic radii for ammonium (NH_4_^+^) is 1.46 Å, methylammonium (MA^+^) is 2.16 Å, formamidinium (FA^+^) is 2.53 Å and ethylammonium (EA^+^) is 2.74 Å. Using these radii, and the revised B cation radii of Table 1, the hybrid iodide perovskites are plotted on the structural map in Fig. 3. It can be seen that due to the larger size of the molecular A site cations, the position of the MA^+^, FA^+^ and EA^+^ compounds lie to the right (high tolerance factor) side of the inorganic analogues – the octahedral factor is of course unchanged as it is not dependent on the A site cation. The Pb(ii) and Sn(ii) compounds fit well into the stability limits of the inorganic perovskites. MAPbI_3_ and MASnI_3_ have tolerance factors of 0.95 and 0.97 respectively. Both these compounds form perovskites, and in fact, MASnI_3_ is the only known iodide compound to form in the undistorted cubic perovskite structure at room temperature and pressure. This correlates well with its tolerance factor being the closest to 1 of any iodide compound, inorganic or hybrid, considered here. FAPbI_3_ and FASnI_3_ have tolerance factors of 1.03 and 1.06 using the revised ionic radii. Both these compounds form perovskites, indicating that, as with the fluoride perovskites,^8^ tolerance factors above 1 can result in perovskite compounds. EAPbI_3_ and EASnI_3_ have tolerance factors of 1.07 and 1.10 respectively. Neither of these compounds form perovskites, and therefore it appears that the high tolerance factor limit for hybrid iodide perovskites is between 1.06 and 1.07. One compound with A = NH_4_^+^ has been characterised: NH_4_PbI_3_, has a tolerance factor of 0.79 and lies outside the stable region for perovskites in Fig. 3, and indeed this compound does not form a perovskite structure at ambient conditions.^47^

Additionally, several hybrid perovskite compounds with Ge(ii) on the B site have been reported, and these do not fit the stability limits shown in Fig. 3 and described above. MAGeI_3_, FAGeI_3_ and ACGeI_3_ were synthesised by Kanatzidis *et al.*^45^ The latter compound includes the acetamidinium CH_3_C(NH_2_)_2_^+^ cation, the radius of which has not been previously calculated using the procedure of Cheetham *et al.* The crystal structure of acetamidinium chloride indicates each C–N bond is 1.305 Å,^48^ and making the simplification of considering the centre of mass to be located at the central carbon atom, we can take *r*_mass_ = 1.305 Å. According to eqn (4), *r*_Aeff_ for the AC^+^ ion is therefore 2.77 Å, significantly larger than MA^+^ or FA^+^. That MAGeI_3_, FAGeI_3_ and ACGeI_3_ form perovskites is surprising, given the octahedral factor significantly smaller than the geometric limit of 0.41, and, in addition, the perovskite ACGeI_3_ has a very large tolerance factor of 1.17. All of the aforementioned Ge(ii) compounds reported by Kanatzidis *et al.* display highly distorted GeI_6_ octahedra, with three short and three much longer Ge–I bonds.^45^ This is attributed by the authors to a stereoactive lone pair on the Ge(ii) centre, and may explain how, in this series of compounds, the octahedral factor and tolerance factor requirements can be relaxed. Only group 14 metals may have stereochemically active lone pairs in the +2 state, meaning this phenomenon will not be widespread and might be seen as an exception to the established stability rules. It may also be that these Ge(ii) compounds, which are formed using low temperature routes, are kinetic products and more thermodynamically stable configurations are possible.

From the results above, we tentatively assign a limit to the stable perovskite region at *t* ≤ 1.06. Whilst the stability limits for the iodide perovskites could be defined with some confidence due to the relatively large number of compounds available, any such limits applied to the hybrid perovskites must be less certain due to the smaller number of compounds available to test the model, as well as the challenge the hybrid structure presents to the assumption of the hard sphere model.

The unusual stability of the Ge(ii) compounds may be related solely to the ability of Ge(ii) to adopt highly distorted coordination due to a stereoactive lone pair. Thus, leaving aside possible Ge(ii) compounds, which do not seem to be predictable using the methods employed here, we show in Table 3 our predictions of unreported compounds that will be stable perovskites, all of which have *μ* > 0.41 and *t* ≤ 1.06. We consider only the NH_4_^+^, MA^+^, FA^+^, AC^+^ and EA^+^ cations, as these have been experimentally incorporated into ABI_3_ structures.

**Table 3:** List of unreported hybrid iodide ABI_3_ compounds that fall within the stable region of the structural map

Compound	Tolerance factor, *t*	Octahedral factor, *μ*
MADyI_3_	0.97	0.44
FADyI_3_	1.06	0.44
MASmI_3_	0.93	0.50
FASmI_3_	1.01	0.50
EASmI_3_	1.05	0.50
ACSmI_3_	1.06	0.50
MATmI_3_	0.98	0.43
FATmI_3_	1.06	0.43
MAYbI_3_	0.98	0.43
MACaI_3_	0.99	0.42
MASrI_3_	0.92	0.53
FASrI_3_	1.00	0.53
EASrI_3_	1.04	0.53
ACSrI_3_	1.05	0.53

Interestingly, several EA and AC compounds are predicated to be stable as perovskites. However, while these compounds may be stable, the B site cations used (Sr^2+^ and Sm^2+^) are likely to have very different contributions to the electronic structure compared with Pb^2+^ and Sn^2+^ that have so far formed the most successful hybrid PV materials. The stability of CH_3_NH_3_SrI_3_ in the perovskite structure has recently been predicted by DFT, in agreement with our analysis here.^28^ However, the band gap is calculated as 3.6 eV, far too high for use as a PV absorber. We have attempted synthesis of MADyI_3_, MAYbI_3_, MATmI_3_ and found the products to be highly moisture sensitive, which has so far precluded a definite structural determination. Whilst isolation of phase pure samples is no doubt possible, it is likely that their instability may prevent technological application in solar cells.

The approach adopted here relates to ABX_3_ hybrid compounds. The related series of alkylammonium metal formates, AB(HCOO)_3_, can be successfully treated by a tolerance factor approach using the traditional Shannon radii.^27^

## Conclusions

We propose an adaptation to the traditional tolerance factor approach for use with halide perovskites in general and hybrid iodide perovskites in particular. Using revised ionic radii, that take into account greater covalency in some metal–halide bonds, and a structure map approach, a system of classification can be devised that can correctly categorise 31 out of 32 inorganic iodide perovskites, and 147 out of 159 ABX_3_ (X = F, Cl, Br, I) compositions. Such a system also seems to apply to the hybrid perovskites, although the Ge(ii) compounds are clear exceptions due to their stereoactive lone pair. We conclude that only a handful of cations may be successfully placed on the B site of an iodide perovskite: Pb, Sn, Yb, Dy, Tm, Sm, Ca, Sr. The Pb and Sn containing hybrid iodide perovskite compounds are very well studied, and we report here that Dy, Tm and Yb hybrid iodide perovskites appear highly moisture sensitive. The Ca and Sr compounds are unlikely to show the narrow band gaps required for PV applications due to the electronic differences between group 2 metals and post transition metals.^28^ There is still more work to do to explore the list of compounds in Table 3, but overall we feel that while there is still great scope for optimisation of existing materials, there may be little opportunity for discovery of entirely new, effective hybrid solar absorber perovskite materials. The search for further hybrid solar absorber materials may therefore have to extend beyond the simple perovskite structure, whether that is towards recently reported double perovskites,^49^ or to more diverse hybrid structures.

## Supplementary Material

SC-007-C5SC04845A-s001
