# Supplementary material for: On the application of the tolerance factor to inorganic and hybrid halide perovskites: a revised system
Source: Chem Sci. 2016 Apr 1;7(7):4548–56. doi: 10.1039/c5sc04845a (PMC6016328; doi:10.1039/c5sc04845a)
Supplement: SC-007-C5SC04845A-s001 [file SC-007-C5SC04845A-s001.pdf]

Electronic Supplementary Material (ESI) for Chemical Science.
This journal is © The Royal Society of Chemistry 2016

Supplementary Information : On the Application of the Tolerance Factor to Inorganic and Hybrid Halide Perovskites: A Revised System

W. Travis, E. N. K. Glover H. Bronstein, D. O. Scanlon and R. G. Palgrave*

Calculation of Ionic Radii

Ionic radii for six coordinate divalent metals in iodide compounds were calculated using crystallographic data from the Inorganic Crystal Structure Database (ICSD) and a general literature search. For a structure to be included the following conditions must be met:

1. The compound must contain a cation in the +2 charge state

2. The metal must be coordinated by exactly six iodide anions and no other anions

3. The structure must be determined at room temperature and pressure

In all suitable structures, the metal-iodide distances for the first coordination sphere of the metal were determined from the crystal structure. The average of these bond lengths was taken for each compound, Table S1 shows the results of this analysis. To calculate the revised ionic radii shown in Tbale 1 of the main manuscript, halide Shannon ionic radius was subtracted from the M-X bond distance.

Bond	Experimental bond length D_{obs} / Å	Standard deviation / Å	Number of compounds used for calculation
Pb-I	3.23	0.06	10
Pb-Br	2.90	0.06	4
Pb-Cl	2.71	0.1	6
Pb-F	2.43	0.03	9
Cr-I	2.88	0.04	4
Cr-Br	2.68	-	1
Cr-Cl	2.53	-	2
Cr-F	2.10	0.01	3
Sn-I	3.17	0.05	7
Mn-I	2.92	0	3
Mn-Br	2.68	0.04	11
Mn-Cl	2.54	0.02	15
Mn-F	2.11	0.01	20
Yb-I	3.12	0.02	4
Yb_Br	2.84	0.07	3
Yb-Cl	2.67	0.07	3
Ca-I	3.12	0.02	3
Ca-Br	2.86	-	2
Ca-Cl	2.73	-	2
Ca-F	2.28	0.05	57
Ni-I	2.77	-	2
Ni-Br	2.54	0.03	5

Ni-Cl	2.41	0.01	13
Ni-F	1.99	0.04	53
Cd-I	3.01	0.03	4
Cd-Br	2.77	0.03	11
Cd-Cl	2.64	0.04	12
Cd-F	2.20	0.1	27
Tm-Cl	2.73	-	1
Tm-I	3.15	0.006	3
Fe-I	2.88	-	2
Fe-Br	2.64	0.04	7
Fe-Cl	2.48	-	2
Fe-F	2.08	0.04	9
Sm-I	3.31	-	1
Sm-Cl	2.82	-	1
Sm-F	2.48	-	2
V-I	2.88	0.03	3
V-Br	2.65	0.02	3
V-Cl	2.51	0.02	9
V-F	2.06	0.05	17
Yb-I	3.13	0.02	4
Yb-F	2.33	0.06	4
Mg-I	2.93	-	2
Mg-Br	2.68	0.02	8
Mg-Cl	2.48	0.04	22
Mg-F	1.98	0.04	143
Ti-I	2.86	0.04	3
Ti-Br	2.66	0.03	7
Ti-Cl	2.53	0.02	11
Hg-I	2.80	0.1	4
Hg-Br	2.72	-	2
Hg-Cl	2.63	0.3	3
Hg-F	2.22	0.07	6
Ge-I	2.97	0.07	4

Table S1: Experimental bond lengths, standard deviations and number of compounds used for calculations.

Compound	Tolerance factor	Octahedral factor
**Perovskites**		
$NaCuF_3$	0.93	0.55
$NaZnF_3$	0.93	0.56
$NaCoF_3$	0.93	0.56
$NaFeF_3$	0.91	0.59
$NaVF_3$	0.91	0.59
$NaCrF_3$	0.90	0.60
$NaMnF_3$	0.89	0.62
$AgCoF_3$	1.01	0.49
$AgNiF_3$	0.99	0.52
$AgMgF_3$	0.97	0.54
$AgZnF_3$	0.96	0.56
$AgMnF_3$	0.92	0.62
$KCoF_3$	1.06	0.49
$KNiF_3$	1.04	0.52
$KMgF_3$	1.02	0.54
$KCuF_3$	1.02	0.55
$KZnF_3$	1.01	0.56
$KFeF_3$	1.00	0.59
KVF_3	0.99	0.59
$KCrF_3$	0.99	0.60
$KMnF_3$	0.97	0.62
$KCdF_3$	0.92	0.71
$KCaF_3$	0.90	0.75
$KHgF_3$	0.89	0.77
$TlCoF_3$	1.08	0.49
$TlCuF_3$	1.04	0.55
$TlFeF_3$	1.02	0.59
$TlCrF_3$	1.01	0.60
$TlMnF_3$	0.99	0.62
$TlCdF_3$	0.94	0.71
$RbCoF_3$	1.09	0.49
$RbCuF_3$	1.05	0.55
$AgCuF_3$	1.05	0.55
$RbZnF_3$	1.04	0.56
$RbFeF_3$	1.02	0.59
$RbVF_3$	1.02	0.59
$RbCrF_3$	1.01	0.60
$RbMnF_3$	1.00	0.62
$RbCdF_3$	0.95	0.71
$RbCaF_3$	0.93	0.75
$RbHgF_3$	0.92	0.77
$RbPbF_3$	0.86	0.89

$CsBeF_3$	1.28	0.34
$CsMgF_3$	1.11	0.54
$CsCdF_3$	1.00	0.71
$CsCaF_3$	0.97	0.75
$CsHgF_3$	0.97	0.77
$CsSrF_3$	0.90	0.89
$CsPbF_3$	0.90	0.89
**Non perovskites**		
$LiMgF_2$	0.85	0.54
$LiZnF_2$	0.84	0.56
$LiMnF_2$	0.81	0.62
$LiCaF_2$	0.75	0.75
$LiPbF_2$	0.69	0.89
$NaCdF_2$	0.84	0.71
$NaCaF_2$	0.83	0.75
$NaPbF_2$	0.76	0.89
$NaBaF_2$	0.72	1.02
$AgPbF_2$	0.79	0.89
$KBaF_2$	0.78	1.02
$RbBeF_2$	1.21	0.34
$RbSrF_2$	0.86	0.89
$CsMnF_2$	1.05	0.62
$CsBaF_2$	0.85	1.02
**Compound**	**Tolerance factor**	**Octahedral factor**
**Perovskites**		
$KFeCl_3$	0.98	0.37
$KMnCl_3$	0.96	0.40
$KCdCl_3$	0.93	0.45
$KCaCl_3$	0.89	0.51
$TlMnCl_3$	0.98	0.40
$TlCdCl_3$	0.94	0.45
$RbCrCl_3$	0.99	0.40
$RbMnCl_3$	0.98	0.40
$RbCdCl_3$	0.95	0.45
$RbCaCl_3$	0.91	0.51
$CsMnCl_3$	1.03	0.40
$CsCdCl_3$	0.99	0.45
$CsCaCl_3$	0.96	0.51
$CsSnCl_3$	0.95	0.52
$CsPbCl_3$	0.93	0.55
**Non perovskites**		
$LiNiCl_3$	0.86	0.33
$LiMgCl_3$	0.84	0.37
$LiVCl_3$	0.83	0.39
$LiCrCl_3$	0.82	0.40

$CuCdCl_3$	0.79	0.45
$CuCaCl_3$	0.76	0.51
$LiCaCl_3$	0.76	0.51
$LiPbCl_3$	0.74	0.55
$NaNiCl_3$	0.94	0.33
$NaCrCl_3$	0.89	0.40
$NaMnCl_3$	0.89	0.40
$NaCdCl_3$	0.86	0.45
$NaCaCl_3$	0.83	0.51
$NaSnCl_3$	0.82	0.52
$NaPbCl_3$	0.81	0.55
$AgMgCl_3$	0.94	0.37
$AgCaCl_3$	0.85	0.51
$AgSnCl_3$	0.80	0.61
$KNiCl_3$	1.01	0.33
$KMgCl_3$	0.98	0.37
$TlMgCl_3$	1.00	0.37
$TlPbCl_3$	0.89	0.55
$RbMgCl_3$	1.00	0.37
$RbFeCl_3$	1.01	0.37
$CsNiCl_3$	1.08	0.33
$CsMgCl_3$	1.05	0.37
$CsFeCl_3$	1.05	0.37
$CsVCl_3$	1.04	0.39
**Compound**	**Tolerance factor**	**Octahedral factor**
**Perovskites**		
$RbCdBr_3$	0.94	0.41
$CsCdBr_3$	0.98	0.41
$CsSnBr_3$	0.94	0.48
$CsPbBr_3$	0.92	0.50
**Non perovskites**		
$LiMgBr_3$	0.81	0.37
$LiCaBr_3$	0.76	0.46
$LiPbBr_3$	0.74	0.50
$NaMgBr_3$	0.88	0.37
$NaCdBr_3$	0.86	0.41
$NaCaBr_3$	0.83	0.46
$AgCdBr_3$	0.88	0.41
$AgPbBr_3$	0.83	0.50
$RbNiBr_3$	1.02	0.30
$RbMnBr_3$	0.97	0.37
$CsNiBr_3$	1.07	0.30
$CsMnBr_3$	1.01	0.37

$CsTiBr_3$	1.02	0.36
**Compound**	**Tolerance factor**	**Octahedral factor**
**Perovskites**		
$CsPbI_3$	0.89	0.47
$CsYbI_3$	0.92	0.43
$CsDyI_3$	0.91	0.44
$CsSnI_3$	0.91	0.44
$CsCaI_3$	0.93	0.42
$CsTmI_3$	0.92	0.44
$RbTmI_3$	0.88	0.43
$RbDyI_3$	0.88	0.44
**Non Perovskites**		
$RbSnI_3$	0.87	0.44
$RbPbI_3$	0.86	0.47
$CsCrI_3$	1.00	0.32
$RbCrI_3$	0.96	0.32
$CsMnI_3$	0.99	0.33
$CsNiI_3$	1.04	0.26
$TlMnI_3$	0.94	0.33
$RbGeI_3$	0.96	0.36
$TlGeI_3$	0.92	0.36
$CsVI_3$	0.99	0.31
$CsMgI_3$	0.98	0.33
$CsTiI_3$	1.00	0.30
$RbVI_3$	0.95	0.31
$RbTiI_3$	0.96	0.30
$CsSrI_3$	0.86	0.53
$RbCaI_3$	0.89	0.41
$KCaI_3$	0.88	0.41
$TlFeI_3$	0.96	0.30
$TlCdI_3$	0.92	0.37
$KTmI_3$	0.86	0.43
$CsGeI_3$	0.92	0.36
$KTiI_3$	0.95	0.30
$TlPbI_3$	0.85	0.47
$RbYbI_3$	0.88	0.43

Table S2: Tolerance and octahedral factors calculated using the revised anion dependent cation ionic radii. Those listed under ‘perovskite’ experimentally form perovskites at room temperature and pressure.

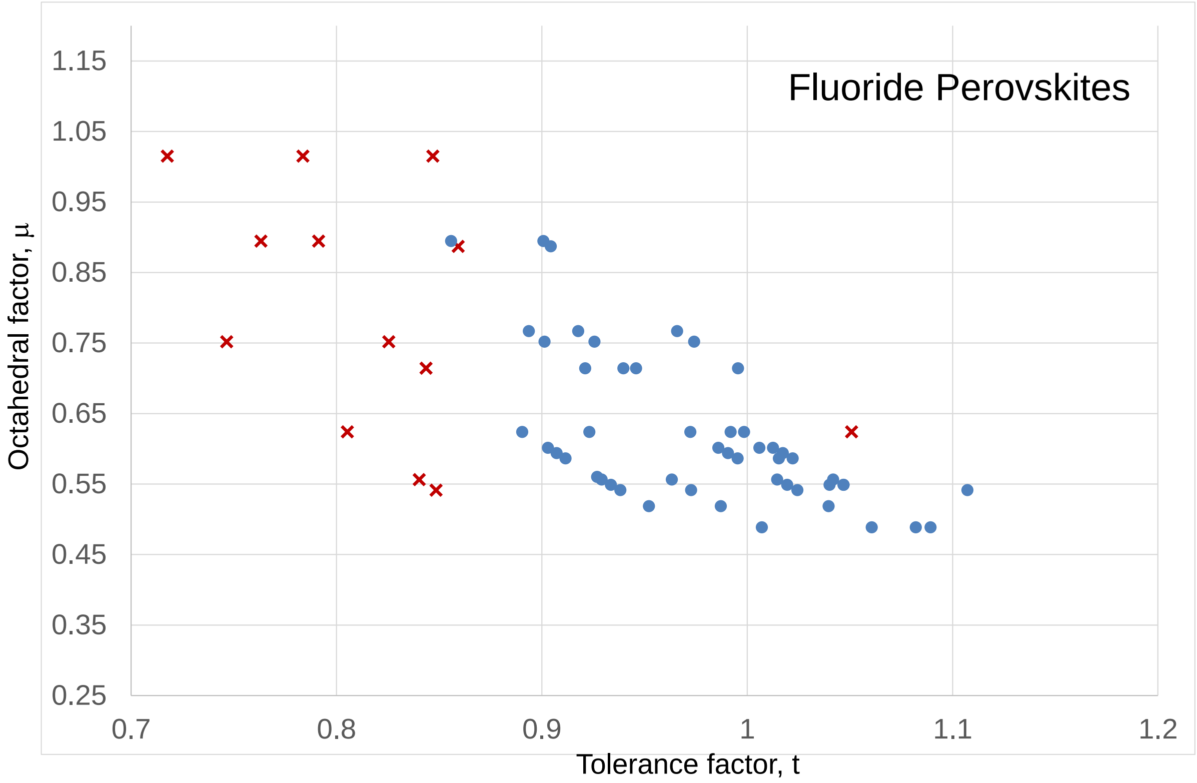

Figure S1. Structural map of AMF_3 compounds. Blue dots represent inorganic compositions that adopt the perovskite structure at room temperature and pressure. Red crosses represent inorganic compounds that do not form perovskites.

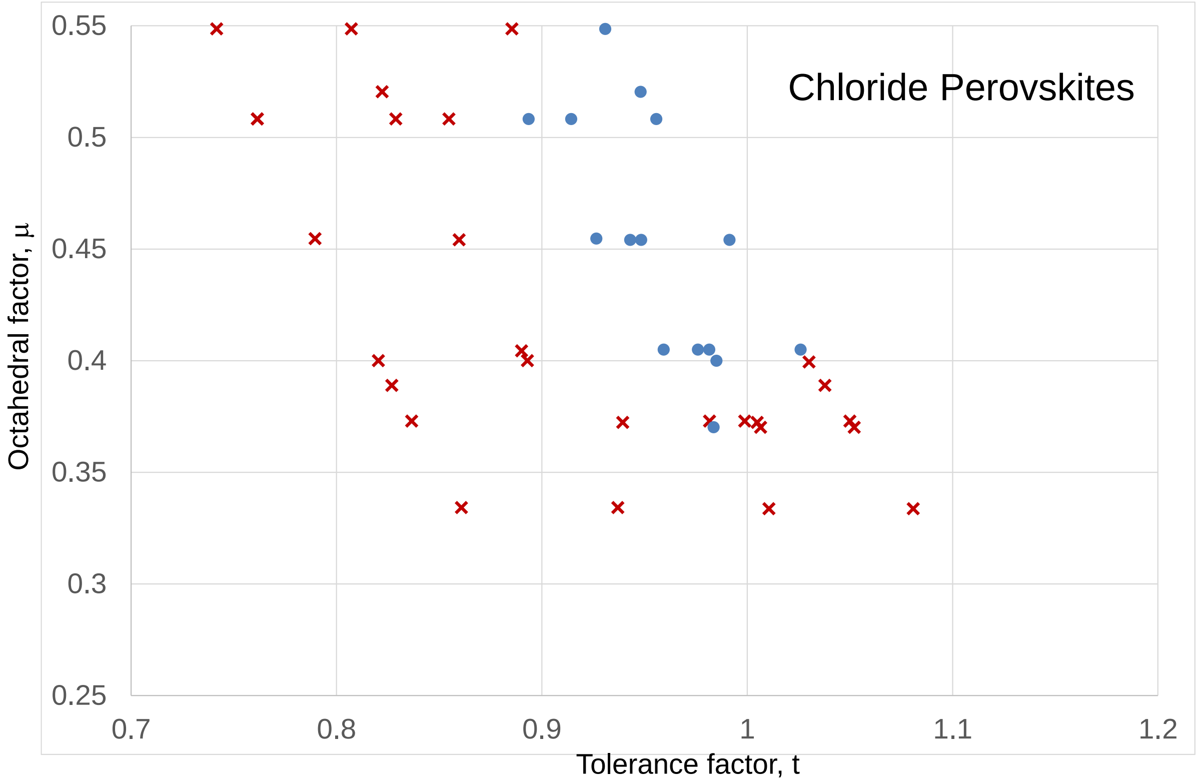

Figure S2. Structural map of $AMCl_3$ compounds. Blue dots represent inorganic compositions that adopt the perovskite structure at room temperature and pressure. Red crosses represent inorganic compounds that do not form perovskites.

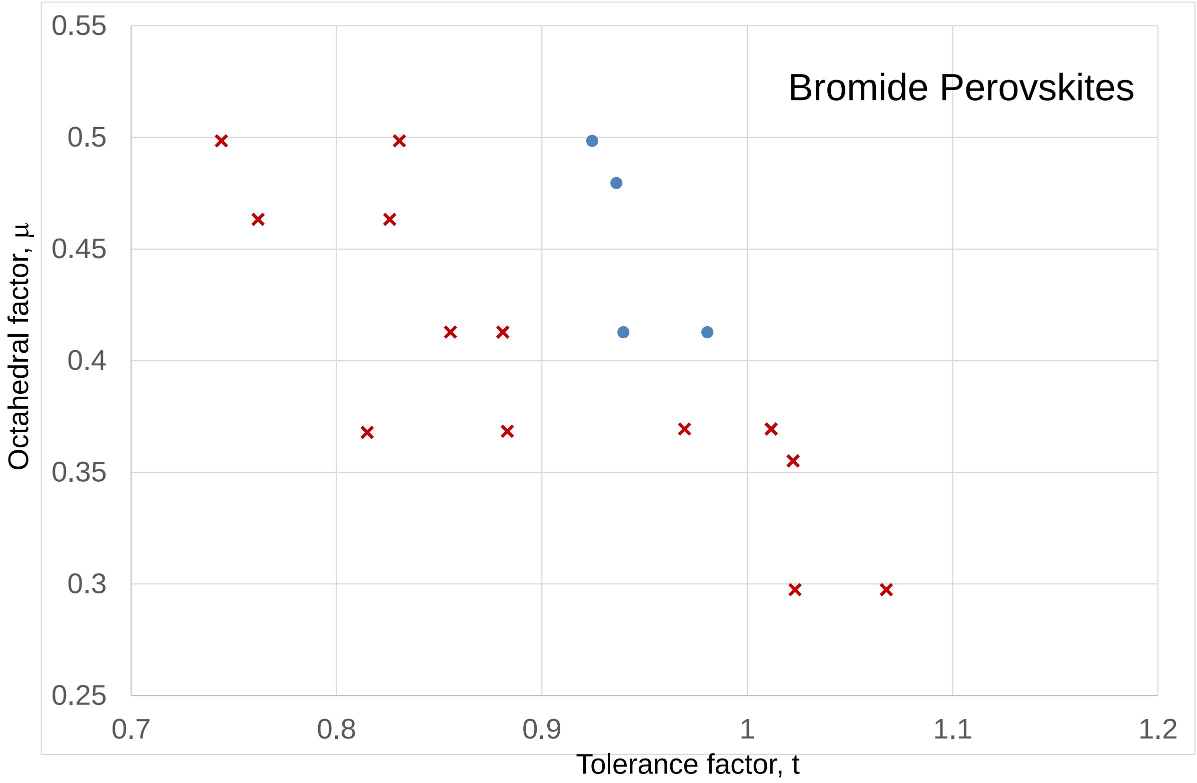

Figure S3. Structural map of $AMBr_3$ compounds. Blue dots represent inorganic compositions that adopt the perovskite structure at room temperature and pressure. Red crosses represent inorganic compounds that do not form perovskites.

	Pb	Sn	Ge	Cr	Mn	Fe	Cd	Ti	V	Ni	Ca	Sr	Mg	Yb	Tm	Dy
**K**											1				2	
**Rb**	3	4	5	6		7		8	8		9			10	2	11
**Cs**	3, 12, 13	12, 14	5	16, 17	17, 18				8	19	9, 20, 21	21	31,32	10		11
**Tl**	22, 23				24	24	7									
**MA**	25	26	27													
**FA**	25	25	27													
**AC**			27													

Table S3. Look up reference table for iodide perovskite papers. A site cation is vertical, B site cation is horizontal. MA = methylammonium, FA = formamidinium, AC = acetamidinium. Reference numbers refer to list below not in main manuscript.

References

1. A. C. Lindsey, M. Zhuravleva, L. Stand, Y. Wu and C. L. Melcher, *Optical Materials*, 2015, **48**, 1-6.
2. G. Schilling, C. Kunert, T. Schleid and G. Meyer, *Zeitschrift Fur Anorganische Und Allgemeine Chemie*, 1992, **618**, 7-12.

3. D. M. Trots and S. V. Myagkota, *Journal of Physics and Chemistry of Solids*, 2008, **69**, 2520-2526.
4. G. Thiele and B. R. Serr, *Zeitschrift Fur Kristallographie*, 1995, **210**, 64-64.
5. G. Thiele, H. W. Rotter and K. D. Schmidt, *Zeitschrift Fur Anorganische Und Allgemeine Chemie*, 1989, **571**, 60-68.
6. L. Guen, R. Marchand, N. Jouini and M. Tournoux, *Revue De Chimie Minerale*, 1978, **15**, 340-345.
7. H. W. Zandbergen, G. C. Verschoor and D. J. W. Ijdo, *Acta Crystallographica Section B-Structural Science*, 1979, **35**, 1425-1427.
8. H. W. Zandbergen, *Journal of Solid State Chemistry*, 1981, **37**, 308-317.
9. E. Beurer, J. Grimm, P. Gerner and H. U. Guedel, *Inorganic Chemistry*, 2006, **45**, 9901-9906.
10. S. H. Wang and M. Zhoa, *Journal of the Less-Common Metals*, 1987, **127**, 219-224.
11. C. Hohnstedt and G. Meyer, *Zeitschrift Fur Anorganische Und Allgemeine Chemie*, 1993, **619**, 1374-1378.
12. C. C. Stoumpos, C. D. Malliakas and M. G. Kanatzidis, *Inorganic Chemistry*, 2013, **52**, 9019-9038.
13. C. K. Moller, *Nature*, 1958, **182**, 1436-1436.
14. I. Chung, J.-H. Song, J. Im, J. Androulakis, C. D. Malliakas, H. Li, A. J. Freeman, J. T. Kenney and M. G. Kanatzidis, *Journal of the American Chemical Society*, 2012, **134**, 8579-8587.
15. G. Thiele, H. W. Rotter and K. D. Schmidt, *Zeitschrift Fur Anorganische Und Allgemeine Chemie*, 1987, **545**, 148-156.
16. L. Guen, N. Jouini and A. Verbaere, *Acta Crystallographica Section B-Structural Science*, 1979, **35**, 1554-1557.
17. G. L. McPherson, L. J. Sindel, H. F. Quarls, C. B. Frederick and C. J. Doumit, *Inorganic Chemistry*, 1975, **14**, 1831-1834.
18. J. P. Sanchez, J. M. Friedt, B. Djermouni and G. Jehanno, *Journal of Physics and Chemistry of Solids*, 1979, **40**, 585-590.
19. A. D. Raw, J. A. Ibers and K. R. Poeppelmeier, *Journal of Solid State Chemistry*, 2012, **192**, 34-37.
20. M. Zhuravleva, B. Blalock, K. Yang, M. Koschan and C. L. Melcher, *Journal of Crystal Growth*, 2012, **352**, 115-119.
21. M. Suta and C. Wickleder, *Journal of Materials Chemistry C*, 2015, **3**, 5233-5245.
22. M. Kocsis, Ieee, Ieee and Ieee, in *1999 Ieee Nuclear Science Symposium - Conference Record, Vols 1-3*, Ieee, New York, 1999, pp. 741-743.
23. W. Stoeger, *Z.Naturforsch.(B)*, 1977, **32**, 975-981.
24. H. W. Zandbergen, *Journal of Solid State Chemistry*, 1981, **37**, 189-203.
25. D. B. Mitzi, in *Progress in Inorganic Chemistry, Vol 48*, ed. K. D. Karlin, John Wiley & Sons Inc, New York, 1999, vol. 48, pp. 1-121.
26. D. B. Mitzi, C. A. Feild, Z. Schlesinger and R. B. Laibowitz, *Journal of Solid State Chemistry*, 1995, **114**, 159-163.
27. C. C. Stoumpos, L. Frazer, D. J. Clark, Y. S. Kim, S. H. Rhim, A. J. Freeman, J. B. Ketterson, J. I. Jang and M. G. Kanatzidis, *Journal of the American Chemical Society*, 2015, **137**, 6804-6819.
